# Minimum fertilization at the appearance of the first flower benefits cotton nutrient utilization of nitrogen, phosphorus and potassium

**DOI:** 10.1038/s41598-020-63692-3

**Published:** 2020-04-22

**Authors:** Honghai Luo, Qiang Wang, Jiekun Zhang, Leishan Wang, Yabing Li, Guozheng Yang

**Affiliations:** 1 College of Plant Science and Technology, Huazhong Agricultural University, 430070 Wuhan, China; 2 Key Laboratory of Oasis Eco-Agriculture, Xinjiang Production and Construction Group, Shihezi University, 832003 Shihezi, Xinjiang China; 3 Cotton Research Institute of the Chinese Academy of Agricultural Science, State Key Laboratory of Cotton Biology, 455000 Anyang, China

**Keywords:** Plant physiology, Environmental sciences

## Abstract

There are currently many problems related to excessive fertilizer application, low fertilizer-use efficiency and lack of an agricultural labor force for cotton production in China. Therefore, the objective of this paper was to explain the optimal application time for once fertilization based on cotton nutrient accumulation of nitrogen, phosphorus and potassium to provide technical support for simplified fertilization management in cotton production. A 2 yr field experiment and a 1 yr pot experiment were conducted with fertilizer (225, 67.5, and 225 kg ha^−1^ of N, P_2_O_5_, and K_2_O, respectively) applied once at 0 (FT1), 5 (FT2), 10 (FT3), 15 (FT4), or 20 (FT5) d after the appearance of the first flower and a triple application (preplant 30%, first bloom 40%, and peak bloom 30%) as the conventional control (FT6). The results showed that FT1 exhibited the greatest nutrient accumulation speed for both the average (5.81, 1.22, and 5.74 kg ha^−1^ of N, P_2_O_5_, and K_2_O, respectively) and the maximum (6.31, 1.44, and 6.24 kg ha^−1^ of N, P_2_O_5_, and K_2_O, respectively) during the fast accumulation period. Moreover, among the different treatments, FT1 exhibited the greatest nutrient recovery and partial productivity. The results suggest that applying the minimum amount of fertilizer at the appearance of the first flower is optimal for maximizing nutrient utilization while minimizing environmental disturbance.

## Introduction

Cotton (*Gossypium hirsutum* L.) is one of the most important fiber-producing crop species worldwide. Increases in the population have led to an increased demand for food and fiber, and threats due to climate change are challenging cotton production^1^. Fertilizer plays a key role in cotton production. During the past half-century, excessive or imbalanced nutrient application has at times resulted in severe environmental problems such as eutrophication^2^, increased greenhouse gas emissions^3^, and soil acidification^4^. Thus, fertilizer efficient utilization by reducing nutrient loss is a crucial environmental issue in the 21st century^5^.

Once fertilizer application constitute a practical, cost-saving, and environmentally sound technique in crop production^6,7^. Compared with conventional fertilizer application practices, minimum applications of slow/controlled-release fertilizers have been shown to increase the yield and nitrogen-use efficiency (NUE) of maize as well as both wheat and rice significantly by 3.1–31.7% and 6.2–86.6%, respectively^8^. The addition of nitrification inhibitors increased grain (wheat, maize and rice) yields and NUE by 6.5–20.1% and 5.0–78.3%, respectively, and significantly reduced nitrous oxide emissions by 22.1–51.0%^9,10^. In cotton production, once fertilizer applications at first bloom (the stage at which 50% of plants presented flowers) reduced labor costs without reducing yields^6^. Liu *et al*. (2018) and Tan and Liu (2018) reported that once fertilizer applications were a viable alternative to multiple split applications because the former resulted in stable crop yields and high fertilizer efficiency and necessitated a smaller labor force; thus, overall, once applications are beneficial for sustainable agricultural production^8,11^.

Plants with a relatively short growth period have the potential to rapidly acquire potassium (K)^12^ and nitrogen (N)^13^ in large amounts. How can the negative impacts be minimized while cotton yields increase or are maintained? One of the keys involves optimizing the timing of once fertilizer application and optimally matching the needs of cotton plants. Hence, we hypothesized that once fertilizer application applied relatively early (i.e., on the day the first flower opened in the field or at the peak squaring stage) is beneficial for improving the accumulation, distribution and use of the main nutrients (and it is also easy for farmers to control fertilizer application timing) and for reducing excessive nutrient losses because of the small plant canopy under the new cropping management system. Therefore, the objective of this study was to verify whether the appearance of the first bloom is the optimal timing for once fertilizer applications in terms of the accumulation, distribution and use of the major nutrients concerning sustainable production.

## Results

### N accumulation

**Figure 1:**
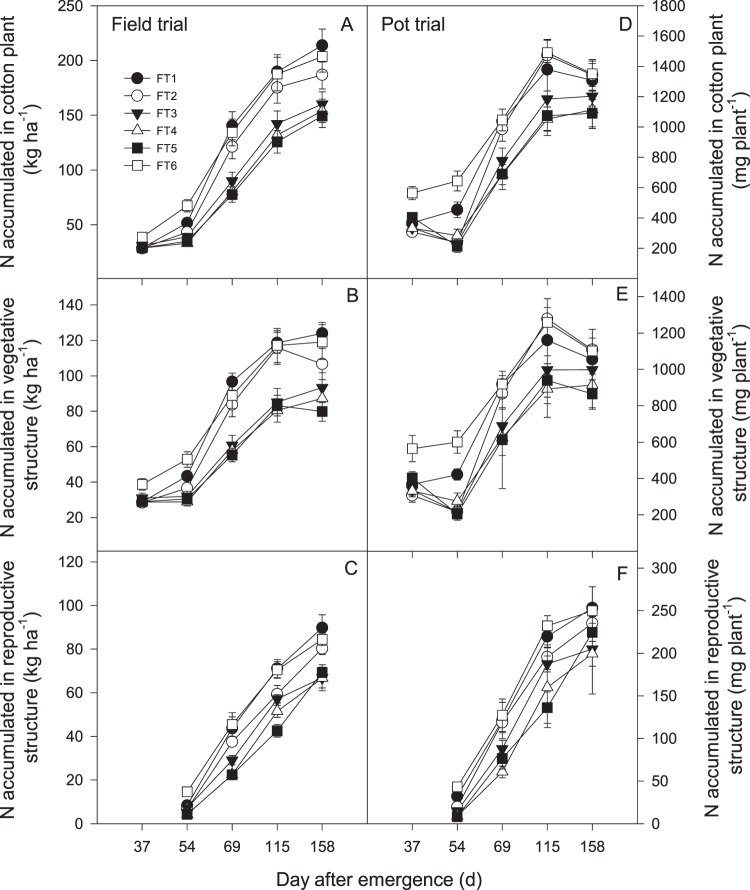
Response of (**A**) cotton plant nitrogen (CPN), (**B**) vegetative structure nitrogen (VSN) and (**C**) reproductive structure nitrogen (RSN) under different once fertilization time in field and pot trial. Error bar shows SE of means. Abbreviations: FT1 = 0 DAF (days after the first flower), FT2 = 5 DAF, FT3 = 10 DAF, FT4 = 15 DAF, and FT5 = 20 DAF and at three splits as the conventional control (FT6) for pre-plant fertilization (30% N, and 100% of the other nutrients), first bloom fertilization (40% N), and peak bloom fertilization (30% N).

The uptake of cotton plant nitrogen (CPN) increased as the plants grew, following a normal sigmoidal curve (Fig. 1A,D). There was no significant effect of once fertilizer application on the N status of plants at squaring (37 DAE), but plant N status was significantly affected during all reproductive phases. FT6 presented the greatest CPN, although there were no differences among the other treatments at 37 or 54 DAE; however, the CPN curves increased to a point at 69 DAE but then flattened for 3 groups (FT1 and FT6 > FT2 > FT3, FT4, and FT5 in the field trial and FT6, FT2, and FT1 > FT3 > FT4 and FT5 in the pot trial) after 115 DAE.

The uptake of vegetative structure nitrogen (VSN) increased as the plant transitioned from one growth phase to another. The VSN was also significantly affected by changes in the fertilizer application time during all growth phases except squaring. FT6 presented the greatest VSN among the treatments at 37 and 54 DAE (Fig. 1B,E). The treatments could be grouped into 2 groups (FT1, FT6, and FT2 > FT3, FT4, and FT5) at 69 DAE and 115 DAE. The maximum VSN was observed in FT1 after 69 DAE in the field trial, but FT6 presented the greatest values throughout the growth period in the pot trial. However, there were no significant differences in VSN between FT1 and FT6.

Reproductive structure nitrogen (RSN) accumulated linearly (Fig. 1C,F). The RSN decreased as the timing of the once fertilizer application was delayed, and the difference increased as the plants grew before maturity. FT6 maintained the greatest RSN until 115 DAE. Thereafter, the RSN in FT1 increased rapidly to peak at 158 DAE in the field trial. FT6 presented the greatest values throughout the growth stage in the pot trial, but there was no significant difference in RSN between FT1 and FT6.

### Simulation of N accumulation

The simulation of biomass accumulation with increasing DAE, which followed a normal logistic growth pattern, was calculated via formula (1). The coefficients of determination were significant because all *P* values were <0.05, with some variation detected in the equation coefficients among the treatments (Table 1).

**Table 1:** Characteristics of cotton N accumulation as varied from different fertilization schedules base on field trial (2012–2013).

Trt	Regression equation	*P* value	Fast accumulation period				
			t_1_(DAE)	t_2_(DAE)	∆t(d)	V_T_(mg d^−1^ p^−1^)	V_M_(mg d^−1^ p^−1^)
**Cotton plant**							
FT1	Y = 215.0232/(1+7.5623e^−0.0632t^)	0.0053	56.7	80.8	24.1	5.81	6.31
FT2	Y = 188.5340/(1+5.8221e^−0.0406t^)	0.0198	58.3	84.3	26.0	4.88	5.39
FT3	Y = 162.0064/(1+5.9162e^−0.0420t^)	0.0033	61.1	98.3	37.1	2.95	3.47
FT4	Y = 155.0550/(1+5.4810e^−0.0310t^)	0.0061	61.3	100.8	39.5	2.50	3.03
FT5	Y = 151.9169/(1+5.4104e^−0.0316t^)	0.0001	62.5	108.3	45.8	2.18	2.72
FT6	Y = 206.2834/(1+7.5623e^−0.0427t^)	0.0101	57.0	82.8	25.8	5.09	5.64
AVE			59.5	92.6	33.1	3.90	4.40
**Vegetative structure**							
FT1	Y = 65.6049/(1+6.1838e^−0.0534t^)	0.0091	46.8	67.9	21.1	1.78	2.28
FT2	Y = 57.5175/(1+4.7956e^−0.0460t^)	0.0205	47.2	77.8	30.6	1.08	1.59
FT3	Y = 53.4508/(1+3.9414e^−0.0327t^)	0.0042	49.4	99.1	49.7	0.62	1.14
FT4	Y = 49.9144/(1+3.7049e^−0.0304t^)	0.0223	50.0	110.2	60.2	0.47	1.00
FT5	Y = 46.2931/(1+4.6205e^−0.0300t^)	0.0003	49.9	111.0	61.1	0.44	0.94
FT6	Y = 63.1846/(1+5.3409e^−0.0605t^)	0.0098	44.5	74.6	30.1	1.21	1.73
AVE			48.0	90.1	42.1	0.90	1.40
**Reproductive structure**							
FT1	Y = 150.1831/(1+8.7584e^−0.0765t^)	0.0018	68.0	88.5	20.5	4.96	5.47
FT2	Y = 131.8226/(1+7.8384e^−0.0522t^)	0.0359	69.6	92.8	23.2	4.05	4.55
FT3	Y = 109.2168/(1+8.8721e^−0.0625t^)	0.0039	77.0	114.1	37.1	2.13	2.65
FT4	Y = 106.1848/(1+7.9634e^−0.0619t^)	0.0087	87.6	125.2	37.6	1.87	2.40
FT5	Y = 105.6440/(1+7.5241e^−0.0513t^)	0.0005	90.5	132.0	41.5	1.76	2.30
FT6	Y = 133.3643/(1+7.2837e^−0.0499t^)	0.0199	66.0	89.6	23.6	4.00	4.50
AVE			76.5	107.0	30.6	3.10	3.60

Where t_1_ and t_2_, mean the beginning and termination day of the fast accumulation period (FAP), respectively. T means the duration of FAP, T = t_2_ − t_1_. V_T_ and V_M_ mean the average and maximum biomass accumulation speed during the FAP, respectively. Abbreviations: FT1 = 0 DAF (days after the first flower), FT2 = 5 DAF, FT3 = 10 DAF, FT4 = 15 DAF, and FT5 = 20 DAF and at three splits as the conventional control (FT6) for pre-plant fertilization (30% N, and 100% of the other nutrients), first bloom fertilization (40% N), and peak bloom fertilization (30% N).

The CPN calculated via formulas (2–4) indicated that the starting and ending days of the 33 d FAP for CPN were 60 DAE and 93 DAE, respectively, when averaged across treatments in the field trial. The average maximum speed (V_M_) was greater than the average speed (V_T_), with varying trends among the different treatments. The FAP in FT1 began the earliest—at 57 DAE; the FAP ended at 81 DAE and persisted for 24 d, with the greatest V_T_ (5.81 kg ha^−1^) and V_M_ (6.31 kg ha^−1^). The FAP in FT5 began and ended at 63 DAE and 108 DAE, respectively, and persisted for 46 DAE; compared with those in the other treatments, the V_T_ and V_M_ in FT5 were minimal.

On average, across all treatments, the fast accumulation period (FAP) of the VSN began and ended at 42 DAE—9 d later than that of its counterpart CPN (Table 1). The V_T_ and V_M_ during the FAP of the VSN were at least two times lower than those of the CPN and occurred earlier. The FAP of the VSN in FT1 began the earliest—at 47 DAE—and ended at 68 DAE, which differed from the other treatments. Compared with the other treatments, FT5 had the longest FAP (61 d). However, FT1 exhibited the greatest V_M_ and V_T_.

Furthermore, compared with that of its counterpart VSN, the FAP of the RSN began 29 d later and ended 17 d later, again with a greater V_M_ and V_T_. The FAP in FT1 and FT6 began the earliest—at 66–68 DAE—and ended at 89–90 DAE. Moreover, compared with the other treatments, FT1 exhibited the shortest FAP (21 d) but the greatest V_M_ and V_T_.

### P accumulation

**Figure 2:**
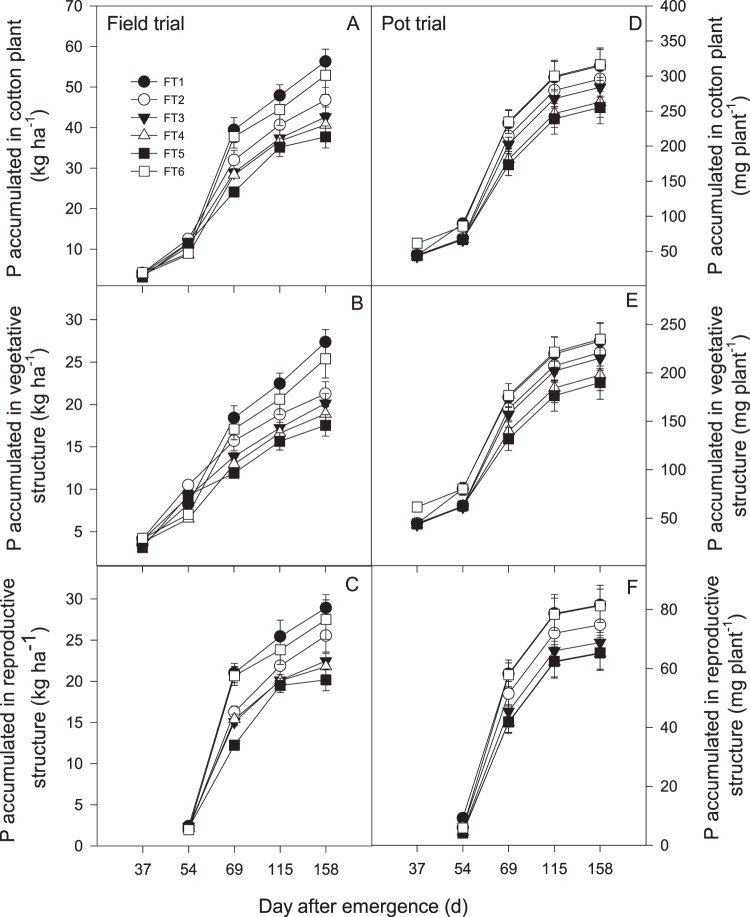
Response of (**A,D**) cotton plant phosphorus (CPP), (**B,E**) vegetative structure phosphorus (VSP), and (**C**,**F**) reproductive structure phosphorus (RSP) under different once fertilization time in field and pot trial. Error bar shows SE of means. Abbreviations: FT1 = 0 DAF (days after the first flower), FT2 = 5 DAF, FT3 = 10 DAF, FT4 = 15 DAF, and FT5 = 20 DAF and at three splits as the conventional control (FT6) for pre-plant fertilization (30% N, and 100% of the other nutrients), first bloom fertilization (40% N), and peak bloom fertilization (30% N).

Cotton plant phosphorus (CPP) increased as the plants grew, following a normal sigmoidal curve, although various trends were detected among the different treatments (Fig. 2). Compared with conventional triple fertilizer application (FT6), once fertilizer application treatments significantly affected CPP accumulation. In addition, compared with the other treatments, FT1 presented the greatest CPP after 115 DAE in the first season. However, in the pot trial, the plants in FT6 and FT1 displayed the greatest growth, with a sharp increase from 69 to 158 DAE. As the plants matured, the CPP curve increased but reached an inflection point at 54 DAE, after which it flattened. There were significant differences among the treatments at various stages, and a normal growth curve was observed.

Vegetative structure phosphorus (VSP) accumulated in a parabolical way, with some variation detected among the treatments. The VSP increased and reached an inflection point at 54 DAE, after which it flattened and increased with maturity in both growing seasons. Compared with that in the other treatments, the maximum VSP in FT1 was detected after 69 DAE in the field trial, but FT6 exhibited the greatest values throughout the growth stage in the pot trial. However, there was no significant difference in VSP between FT1 and FT6.

The cotton plants showed a different trend in terms of the accumulation of reproductive structure phosphorus (RSP). The organs had not developed until 54 DAE across both growing seasons (Fig. 2). As the plants matured, differences became more evident among the different treatments, particularly in the field trial. FT5 exhibited the lowest values throughout the growing season, and FT1 exhibited the greatest values in the field trial. Moreover, FT6 exhibited the greatest values throughout the growth stage in the pot trial, but there was no significant difference in RSP between FT1 and FT6.

### Simulation of P accumulation

The simulation of P accumulation with cotton growth stage was calculated via formula (1). The logistic function of P accumulation followed a normal sigmoidal pattern because all *P* values were <0.05 (Table 2).

**Table 2:** Characteristics of cotton P accumulation as varied from different fertilization schedules base on field trial (2012–2013).

Trt	Regression equation	P value	Fast accumulation period				
			t_1_(DAE)	t_2_(DAE)	∆t(d)	V_T_(mg d^−1^ p^−1^)	V_M_(mg d^−1^ p^−1^)
**Total plant**							
FT1	Y = 58.0232/(1+7.5623e^−0.0632t^)	0.0053	61.0	88.7	27.7	1.22	1.44
FT2	Y = 48.5340/(1+5.8221e^−0.0406t^)	0.0198	62.6	92.2	29.7	0.95	1.17
FT3	Y = 44.0064/(1+5.9162e^−0.0420t^)	0.0033	63.3	99.8	36.5	0.70	0.92
FT4	Y = 42.0550/(1+5.4810e^−0.0310t^)	0.0061	63.6	101.4	37.8	0.65	0.87
FT5	Y = 39.9169/(1+5.4104e^−0.0316t^)	0.0001	64.8	106.0	41.2	0.56	0.79
FT6	Y = 55.2834/(1+7.5623e^−0.0427t^)	0.0101	61.2	91.0	29.7	1.07	1.30
AVE			62.7	96.5	33.7	0.90	1.10
**Vegetative structure**							
FT1	Y = 19.6049/(1+6.1838e^−0.0534t^)	0.0091	51.1	75.8	24.7	0.47	0.69
FT2	Y = 16.5175/(1+4.7956e^−0.0460t^)	0.0205	51.4	85.5	34.0	0.30	0.52
FT3	Y = 16.4508/(1+3.9414e^−0.0327t^)	0.0042	51.5	91.4	39.9	0.23	0.46
FT4	Y = 15.9144/(1+3.7049e^−0.0304t^)	0.0223	52.3	99.7	47.4	0.20	0.42
FT5	Y = 15.2931/(1+4.6205e^−0.0300t^)	0.0003	52.2	100.5	48.3	0.18	0.41
FT6	Y = 18.1846/(1+5.3409e^−0.0605t^)	0.0098	48.7	82.5	33.8	0.31	0.53
AVE			51.2	89.2	38.0	0.30	0.50
**Reproductive structure**							
FT1	Y = 39.1831/(1+8.7584e^−0.0765t^)	0.0018	72.3	93.8	21.6	1.05	1.27
FT2	Y = 32.8226/(1+7.8384e^−0.0522t^)	0.0359	73.9	100.6	26.7	0.72	0.94
FT3	Y = 28.2168/(1+8.8721e^−0.0625t^)	0.0039	79.1	116.6	37.5	0.43	0.66
FT4	Y = 27.1848/(1+7.9634e^−0.0619t^)	0.0087	89.9	127.8	37.9	0.41	0.64
FT5	Y = 24.6440/(1+7.5241e^−0.0513t^)	0.0005	92.8	131.7	38.9	0.37	0.60
FT6	Y = 37.3643/(1+7.2837e^−0.0499t^)	0.0199	72.2	95.5	25.3	0.85	1.07
AVE			80.0	111.0	31.3	0.60	0.90

Where t_1_ and t_2_, mean the beginning and termination day of the fast accumulation period (FAP), respectively. T means the duration of FAP, T = t_2_ − t_1_. V_T_ and V_M_ mean the average and maximum biomass accumulation speed during the FAP, respectively. Abbreviations: FT1 = 0 DAF (days after the first flower), FT2 = 5 DAF, FT3 = 10 DAF, FT4 = 15 DAF, and FT5 = 20 DAF and at three splits as the conventional control (FT6) for pre-plant fertilization (30% N, and 100% of the other nutrients), first bloom fertilization (40% N), and peak bloom fertilization (30% N).

The CPP calculated from formulas (2–4) indicated that the starting and ending days of the 34 d FAP for CPP were 63 and 97 DAE, respectively, averaged across treatments in the field trial. The FAP in FT1 began the earliest—at 61 DAE—and ended at 89 DAE. Furthermore, both the average (1.22 kg ha^−1^) and maximum (1.44 kg ha^−1^) CPP accumulation rates in the plants in the FT1 treatment were greater than those in the plants in the other treatments. The FAP in FT5 began and ended the latest—at 65 DAE and 106 DAE, respectively—and persisted for 41 DAE, with the lowest V_T_ and V_M_ among all the treatments.

Changes in the timing of the once fertilizer application also influenced the progression of VSP accumulation. Among all the treatments, the FAP of the VSP in FT6 began the earliest—at 49 DAE. However, the FAP of VSP in FT5 ended the latest—at 101 DAE—and had the longest duration (48 d). Moreover, FT1 was superior to the other treatments in terms of the V_T_ and V_M_ of the FAP (0.47 kg ha^−1^ and 0.69 kg ha^−1^, respectively).

Averaged across treatments, the FAP of the RSP uptake began at 80 DAE and ended at 110 DAE. Compared with that in the other treatments, the RSP accumulation in FT1 exhibited the shortest FAP (22 d) but the greatest average (1.05 kg ha^−1^) and the greatest maximum (1.27 kg ha^−1^) rates. Moreover, the RSP in FT5 had the greatest FAP duration (39 d) and the lowest average (0.37 kg ha^−1^) and lowest maximum (0.60 kg ha^−1^) rates.

### K accumulation

**Figure 3:**
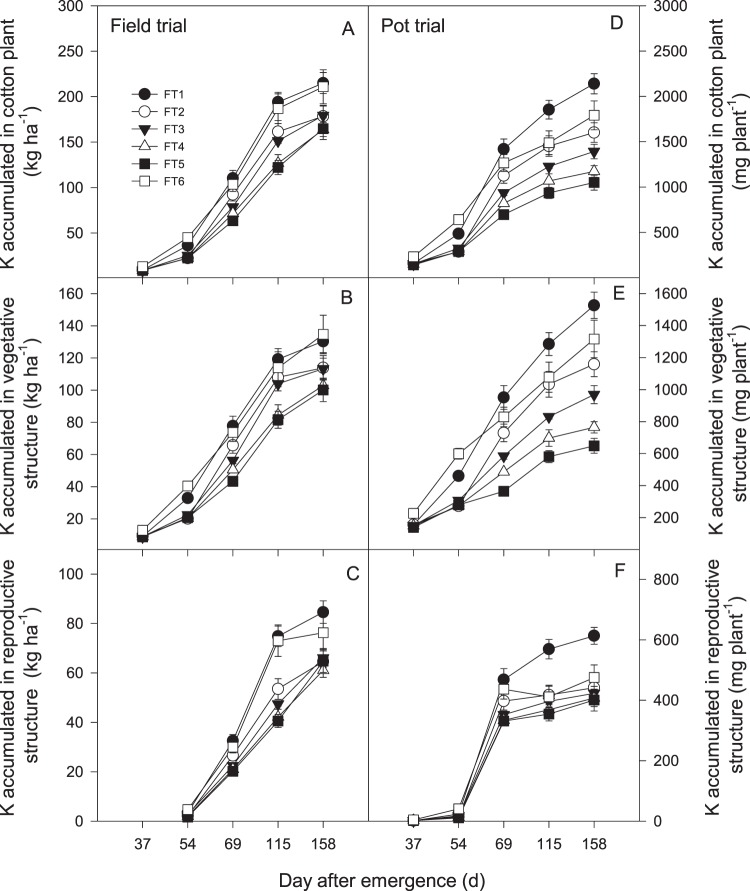
Response of (**A,D**) cotton plant potassium (CPK), (**B,E**) vegetative structure potassium (VSK), and (**C,F**) reproductive structure potassium (RSK) under different once fertilization time in field and pot trial. Error bar shows SE of means. Abbreviations: FT1 = 0 DAF (days after the first flower), FT2 = 5 DAF, FT3 = 10 DAF, FT4 = 15 DAF, and FT5 = 20 DAF and at three splits as the conventional control (FT6) for pre-plant fertilization (30% N, and 100% of the other nutrients), first bloom fertilization (40% N), and peak bloom fertilization (30% N).

Compared with the conventional triple fertilizer application, once fertilizer applications affected cotton plant potassium (CPK) accumulation at later growth stages (Fig. 3). FT1 exhibited the greatest CPK, with no differences detected among the other treatments at 37 or 54 DAE. The treatments could be grouped into 3 groups (FT1 and FT6 > FT2 and FT3 > FT4 and FT5) after 115 DAE in the field trial and 4 groups (FT1 > FT6 and FT2 > FT3 > FT4 and FT5) after 69 DAE in the pot trial.

The accumulation of vegetative structure potassium (VSK) was strongly influenced by once fertilizer applications during both years. The VSK decreased as fertilizer applications were delayed, and the differences increased as the plants matured. The maximum VSK was recorded in FT6 at 158 DAE in the field trial, but there was no significant difference between FT6 and FT1. The levels of cotton VSK in the pot trial were in the following order: FT1 > FT6 and FT2 > FT3 > FT4 > FT5.

FT1 presented the greatest reproductive structure potassium (RSK), with no differences among the other treatments at 37 or 54 DAE. The treatments could be grouped into 2 groups (FT1 and FT6 > FT2, FT3, FT4, and FT5 in the field trial and FT1 > FT6, FT2, FT3, FT4, and FT5 in the pot trial) at 115 DAE and 158 DAE.

### Simulation of K accumulation

According to formulas (2)-(4), the starting and ending days of the FAP of CPK accumulation were 60 and 92 DAE, respectively, averaged across all treatments (Table 3). Delaying the once fertilizer application timing slowed the speed of K accumulation, but the speed was relatively slower in FT6 than in FT1. The CPK uptake during the FAP in terms of the V_T_ and V_M_ was greater in FT1 than in FT6 (5.74 kg ha^−1^ and 6.24 kg ha^−1^, respectively), and the rates were similar to those in FT6 compared with the other treatments.

**Table 3:** Characteristics of cotton K accumulation as varied from different fertilization schedules base on field trial (2012–2013).

Trt	Regression equation	P value	Fast accumulation period				
			t_1_(DAE)	t_2_(DAE)	∆t(d)	V_T_(mg d^−1^ p^−1^)	V_M_(mg d^−1^ p^−1^)
**Cotton plant**							
FT1	Y = 215.0232/(1+6.5623e^−0.0732t^)	0.0053	57.8	83.6	25.8	5.74	6.24
FT2	Y = 188.5340/(1+4.8221e^−0.0456t^)	0.0198	59.3	87.0	27.7	5.35	5.86
FT3	Y = 180.0064/(1+4.9162e^−0.0480t^)	0.0033	60.1	95.7	35.6	4.16	4.68
FT4	Y = 166.0550/(1+4.4810e-^0.0410t^)	0.0061	60.4	98.3	37.9	3.91	4.44
FT5	Y = 163.9169/(1+4.4104e^−0.0356t^)	0.0001	61.6	102.9	41.3	3.58	4.12
FT6	Y = 211.2834/(1+4.5289e^−0.0527t^)	0.0101	58.0	85.6	27.6	5.37	5.92
AVE			59.5	92.2	32.7	4.69	5.21
**Vegetative structure**							
FT1	Y = 74.6049/(1+5.0838e^−0.0634t^)	0.0091	47.9	70.7	22.8	1.88	2.38
FT2	Y = 71.5175/(1+3.7956e^−0.0560t^)	0.0205	48.2	80.5	32.3	1.33	1.84
FT3	Y = 71.4508/(1+2.9414e^−0.0367t^)	0.0042	48.3	96.5	48.2	0.89	1.41
FT4	Y = 57.9144/(1+2.7049e^−0.0324t^)	0.0223	49.1	107.7	58.6	0.73	1.26
FT5	Y = 55.2931/(1+2.6205e^−0.0200t^)	0.0003	49.0	105.6	56.6	0.76	1.26
FT6	Y = 92.1846/(1+4.3409e^−0.0615t^)	0.0098	45.5	77.4	31.9	1.35	1.87
AVE			48.0	89.7	41.7	1.16	1.67
**Reproductive structure**							
FT1	Y = 141.1831/(1+8.3584e^−0.0865t^)	0.0018	69.1	85.7	16.6	5.78	6.29
FT2	Y = 110.8226/(1+7.0384e^−0.0622t^)	0.0359	70.6	95.5	24.9	3.88	4.38
FT3	Y = 109.2168/(1+7.8721e^−0.0725t^)	0.0039	75.9	111.5	35.6	2.71	3.23
FT4	Y = 109.1848/(1+7.2634e^−0.0649t^)	0.0087	86.7	122.7	36.0	2.67	3.20
FT5	Y = 108.6440/(1+6.5241e^−0.0553t^)	0.0005	89.6	126.6	37.0	2.60	3.14
FT6	Y = 113.3643/(1+6.0837e^−0.0529t^)	0.0199	67.0	92.4	25.4	3.79	4.29
AVE			76.5	105.7	29.3	3.57	4.09

Where t_1_ and t_2_, mean the beginning and termination day of the fast accumulation period (FAP), respectively. T means the duration of FAP, T = t_2_ − t_1_. V_T_ and V_M_ mean the average and maximum biomass accumulation speed during the FAP, respectively. Abbreviations: FT1 = 0 DAF (days after the first flower), FT2 = 5 DAF, FT3 = 10 DAF, FT4 = 15 DAF, and FT5 = 20 DAF and at three splits as the conventional control (FT6) for pre-plant fertilization (30% N, and 100% of the other nutrients), first bloom fertilization (40% N), and peak bloom fertilization (30% N).

Compared with that of its counterpart CPK, the FAP of the VSK began 12 d earlier but ended 2 d later (Table 4). Among the different treatments, the progression of VSK accumulation was affected by the timing of fertilizer application. The FAP of the VSK in FT6 began the earliest—at 46 DAE—and presented a maximum rate of 1.87 kg ha^−1^. However, a similar trend was also observed in FT1, with the greatest maximum rate of 2.38 kg ha^−1^ during the FAP.

**Table 4:** Effects of fertilization schedule on nutrient uptake and efficiencies by cotton plant based on field trial (2012–2013).

Treatments	Nutrient recovery ratio (%)			Nutrient partial productivity (kg kg^−1^)		
	N	P	K	N	P	K
FT1	85.0a	73.4a	85.5a	6.28a	20.93a	6.28a
FT2	73.1b	59.3b	69.3b	5.77ab	19.24ab	5.77ab
FT3	61.2c	53.1b	69.7b	5.69ab	18.96ab	5.69ab
FT4	58.4c	50.2b	62.8c	5.11ab	17.04bc	5.11ab
FT5	56.3c	45.9c	63.2c	4.97b	16.56c	4.97b
FT6	80.5a	68.3a	83.7a	6.16a	20.53a	6.16a

Means within a column of the same year followed by a different letter are significantly different (P < 0.05) according to the Duncan multiple range test. Abbreviations: FT1 = 0 DAF (days after the first flower), FT2 = 5 DAF, FT3 = 10 DAF, FT4 = 15 DAF, and FT5 = 20 DAF and at three splits as the conventional control (FT6) for pre-plant fertilization (30% N, and 100% of the other nutrients), first bloom fertilization (40% N), and peak bloom fertilization (30% N).

In FT1, the FAP began at 69 DAE and ended at 86 DAE, and the speed in terms of both the V_T_ and V_M_ was relatively high for the RSK accumulation. Similar trends were detected in FT5, albeit with lower speeds than those observed in FT1.

### Absorption and production efficiency of N, P and K in cotton plants

FT1 and FT6 exhibited similar N, P and K nutrient recovery ratios: the N in FT1 and FT6 was 10.4%, 23.2%, 26.3%, and 28.5% greater than that in FT2-FT5, respectively; the P was 14.3%, 22.0%, 25.5%, and 30.9% greater, respectively; and the K was 16.2%, 15.8%, 23.0%, and 22.6% greater, respectively (Table 4). The nutrient partial productivity (NPP) decreased from FT1 to FT5 when the once fertilizer application timing was delayed, but there were no significant differences in NPP between FT1 and FT6. These results suggest that once fertilizer application at the appearance of the first flower can increase nutrient uptake and productivity, especially for N and K.

## Discussion

### Once fertilizer applications are optimal for the accumulation of major nutrients

Proper nutrient applications that meet but do not exceed crop nutrient requirements are essential for achieving maximum yields while minimizing environmental risk; these applications can greatly enhance root growth and efficiency of the rhizosphere in terms of nutrient mobilization and capture^14,15^. Our previous study showed that once fertilizers applied at the appearance of the first flower reduced labor costs without reducing yields (1396 kg ha^−1^) when the cotton was grown as part of a highly efficient production system that involved late planting, no plastic mulching film, and once fertilizer application at a low rate^16^. For continuous growth, plants need an adequate nutrient supply, and nutrient absorption can vary in quantity and rate during different growth periods^17^. Cotton growth and yield are positively associated with nutrient uptake by roots^18^. In the present study, compared with the triple fertilizer application, the once fertilizer application at the appearance of the first flower exhibited the maximum CPN, CPP and CPK accumulation, which is beneficial for biomass accumulation. This finding was in accordance with that of Lemaire and Gastal (2009)^19^, who reported that plant N uptake rates were closely linked to biomass as plants mature. However, the results of the present study showed that the accumulation of CPN, CPP and CPK was delayed, the FAP was shorter, the speed in terms of both V_T_ and V_M_ was greater, and the subsequent plant nutrient accumulation was greater as the once fertilizer application timing was delayed compared with no delay, indicating that increasing the fertilizer supply at the appearance of the first flower should be beneficial for increasing the speed of cotton plant nutrient accumulation and for accumulating the greatest amount of nutrients in the shortest possible time; thus, nutrient loss is relatively unlikely. This approach would benefit the environment, such as by reducing greenhouse gas emissions and reducing water eutrophication^20^.

The results of the present study also showed that cotton plants treated with a once fertilizer application at the appearance of the first flower accumulated more N, P and K in their reproductive tissues at the boll opening and plant removal stages, especially K in reproductive organs in both the pot and field trials, which resulted in the greatest reproductive biomass and yield of the cotton plants^16^. These results are in accordance with those of Tang *et al*. (2012)^11^, who reported that 79% of ^15^N accumulated in reproductive organs when N fertilizer was applied relatively late. Khan *et al*. (2017b) revealed in a 2 yr experiment that a moderate planting density or early sowing date increased K uptake and that K was allocated to the reproductive organs^13^. These results suggest that more K uptake and allocation to the reproductive organs than to the vegetative organs promoted reproductive organ biomass, which resulted in increased yields. Compared with once fertilizer applications at the appearance of the first flower, those at the appearance of the first bloom have the potential to increase total plant K acquisition but can reduce K allocated to reproductive organs, which can reduce yields.

### Optimal once fertilizer applications for increasing nutrient-use efficiency

In China, the excessive use of chemical fertilizers (especially N fertilizers) has often been considered the main practical strategy for achieving high yields. However, many recent case studies have shown that N fertilizer use in China could often be reduced by half without losses in yield or grain quality, and thus, N losses could be reduced to less than 50%^21–23^. In the present study, under the same fertilizer rate (225 kg ha^−1^ N, 67.5 kg ha^−1^ P_2_O_5_, 225 kg ha^−1^ K_2_O, and 1.5 kg ha^−1^ B), FT1 and FT6 exhibited similar N, P and K nutrient absorption, but this absorption was significantly greater than that in the other treatments. These results indicated that the optimal once fertilizer application time can significantly increase nutrient absorption. Furthermore, Magdoff *et al*. (2000) indicated that farming practices that cause nutrient imbalances can reduce pest resistance^22^. Therefore, once fertilizer applications at the appearance of the first flower can induce cotton plant growth and tolerance to both insect pests and diseases because of the nutrient absorption of the plants.

Zhang *et al*. (2013) reported that financial support to promote the development of a contractor sector for fertilizer applications can be beneficial^5^. Such contractors can (i) purchase machinery for subsurface urea applications, reducing ammonia losses; (ii) apply N at the “right time” overcoming the labor shortage problem; and (iii) compose a professional group that receives technical information on N fertilizer management. The NPP results in the present study further confirmed that among those in all the treatments,the plants in FT1 used more N and K to produce the greatest yield. These findings suggest that once fertilizer applications at the appearance of the first flower should occur with a relatively high nutrient content to improve N and K accumulation and use and possibly increase yields. These actions are crucial to reducing N deposition and its negative impact both locally and globally.

### Optimal once fertilizer applications for sustainable production

Until recently, most agricultural systems in China have focused on increasing production via large inputs of resources, especially water and nutrients, often at the sake of the environment^24^. In 2015, the Chinese government officially launched the “Action Plan for the Zero Increase of Fertilizer Use” (APZIFU). The aim was to stop the increase in synthetic fertilizer use by 2020 without reducing food production^25^. This “zero increase” plan highlights the need to adopt reasonable N management to improve NUE practices, which is a key step in reducing the unintended climate and environmental changes induced by N fertilizer applications^26^. Xia *et al*. (2017) also reported that, compared with traditional N management practices, knowledge-based N practices overall have reduced greenhouse gas emissions and major reactive N losses; specifically, the reductions are 5.4–39.8% for N_2_O emissions, 30.7–61.5% for NH_3_ emissions, 13.6–37.3% for N leaching, and 15.5–45.0% for N runoff^27^. Compared with conventional fertilizer methods, the once fertilizer application technique applied to three major cereal crop species (wheat, rice and corn) could significantly reduce the loss of NH_3_ volatilization, N_2_O emissions, N leaching and runoff by 18.1–81.3%, 22.4–73.4%, 0–53.0% and 0–43.2%, respectively^8^. In the present study, although the greenhouse gas emissions and N losses were not directly measured, the high nutrient recovery and NPP in response to once fertilizer application at the appearance of the first flower are beneficial for economizing cotton production and reducing environmental disturbance. Therefore, optimal nutrient management strategies can significantly reduce fertilizer rates without reducing crop yields^21,28^, with multiple benefits to agriculture and the environment^29^.

## Conclusion

In this study, once fertilizer applications at the appearance of the first flower resulted in the FAP of major nutrients (N, P and K) occurring the earliest, and the shortest duration was maintained with the greatest speed. The nutrient recovery and NPP results also revealed that once fertilizer applications at the appearance of the first flower increased nutrient uptake and productivity, especially for N and K. On the basis of the findings of the present study, it can be concluded that once fertilizer applications at the appearance of the first flower should be the optimal choice to maximize nutrient accumulation, economize cotton production and reduce environmental disturbance.

## Materials and methods

### Site description

This study consisted of field trials in 2012 and 2013 and an open pot trial in 2013 at the Experimental Farm of Huazhong Agricultural University, Wuhan, China (30°37N, 114°21E, 23 m above sea level). The soil was a yellowish brown clay loam and was alkaline, with 110.2 mg kg^−1^ N, 15.3 mg kg^−1^ P_2_O_5_, and 99.9 mg kg^−1^ K_2_O within the 0–20 cm layer (the pot soil was collected from the same field). Over two years, the mean maximum and minimum temperatures were 34.2 °C and 15.3 °C and 35.7 °C and 15.1 °C, with rainfall levels of 1072 mm and 1076 mm, respectively, during the cotton growing season.

### Experimental design and crop management

The cotton (*G. hirsutum* L. cv. Huazamian H318) plants used were high-yielding commercial cultivars. Fertilizer (225 kg ha^−1^ N, 67.5 kg ha^−1^ P_2_O_5_, 225 kg ha^−1^ K_2_O, and 1.5 kg ha^−1^ B) was applied once at five different times (treatments): FT1, 0 DAF (days after the first flower); FT2, 5 DAF; FT3, 10 DAF; FT4, 15 DAF; and FT5, 20 DAF. The fertilizer was also applied as part of a triple application: at preplanting (30% of the N, and 100% of the other nutrients), first bloom (40% of the N), and peak bloom (30% of the N), which constituted the conventional control treatment (FT6). The fertilizers applied included urea (46.3% N), calcium superphosphate (12% P_2_O_5_), potassium chloride (59% K_2_O), and borate (10% B).

In 2013, two holes (5 mm in diameter) were drilled into the bottom of polyvinyl chloride (PVC) pots (40 cm in height, 35 cm in diameter) for leaching. Each pot contained 45 kg of soil (21% water by weight). Four seeds were sown in 4 rows per plot, and 5 pots constituted one treatment, each of which was replicated three times. With respect to the preplant application, the fertilizers and the top 20 cm of soil were mixed evenly at 2 d before sowing. With respect to the other applications, the fertilizer (N) was first dissolved in water (0.4%, wt/wt), which was subsequently applied around the plants 10 cm from the roots. Three seeds were sown per pot on 1 June 2013, and at the one-leaf stage, the seedlings were subsequently thinned to one per pot. The pots were covered when they rained heavily to prevent water lodging or overflowing, and the plants were watered with 2 L in the evening whenever the upper leaves appeared to be wilted before 11 a.m.

The plots, which were 43.2 m^2^ (12 m × 3.6 m) in size were arranged randomly and replicated four times. Four seeds were sown in 4 rows per plot on 17 June 2012 and 23 May 2013. The plants were spaced 10 cm apart, and the rows were spaced 66 cm apart, with a planting density of 75 000 plants ha^−1^. The seedlings were thinned at the three-leaf stage to the required density. Field management practices were conducted in accordance with normal local practices.

### Sampling and measurements

Biomass samples of 9 cotton plants from each treatment were used to determine the N, P and K accumulation at 5 different stages: squaring (37 d after emergence [DAE]), first flowering (54 DAE), peak flowering (69 DAE), boll opening (115 DAE) and plant removal (158 DAE). Each sample was divided into vegetative structures (roots, stems, leaves, fruiting branches) and reproductive organs (squares, flowers, bolls) and then placed in an envelope. The subsamples were then placed in an electric fan-assisted oven for quick heating at 105 °C for 30 min and then dried at 70 °C to a constant weight. The dried samples were subsequently processed with a Wiley mill to pass through a 0.5 mm sieve.

The total N content was determined according to the micro-Kjeldahl method. Approximately 0.2 g of milled sample tissue was digested for 1 h in concentrated H_2_SO_4_ plus ¼-strength catalyst tablets. After they cooled, the digests were alkalinized with 40% NaOH solution, after which distilled NH_3_ was collected and added to 10 ml of boric acid containing an indicator. The total N was determined by titrating the distillate against 0.01 M HCl. The P content was determined colorimetrically via a spectrophotometer, and the K content was assessed via an atomic adsorption spectrophotometer (TAS-990, Beijing, China). The NPK nutrient recovery ratio (%) was determined according to the methods of Moll *et al*. (1982) as follows^30^: Nutrient recovery ratio (%) = Total plant NPK at removal/NPK fertilizer×100. Similarly, the NPK partial productivity (kg kg^−1^) was determined as follows: NPK partial productivity (kg kg^−1^) = cotton seed yield/NPK fertilizer.

### Statistical analysis

The cotton N, P, and K contents were not significantly different between the two growing seasons in the field; therefore, the values presented are the means of 2 yr. Microsoft Excel 2007 and SigmaPlot 12.0 were used for data processing and figure drawing, respectively. SPSS 12.0 was used for performing ANOVA and regression analysis. A logistic formula was used to describe the progression of N, P, and K accumulation as follows^6^:

1 $${\rm{Y}}={\rm{K}}/1+{{\rm{ae}}}^{{\rm{bt}}}$$

Here, *t* (d) represents the DAE; *Y*(g) represents the N, P, and K contents at *t*; *K*(g) represents the maximum content; and *a* and *b* are constants to be retrieved.

From formula (1):

2 $${t}_{0}={\rm{lna}}/{\rm{b}},{t}_{1}=1/{\rm{bln}}(2+\ddot{O}3/{\rm{a}}),{t}_{2}=1/{\rm{bln}}(2-\ddot{O}3/{\rm{a}})$$

When *t* = *t*_0_, the N, P, and K accumulation has a V_M_ calculated as follows:

3 $${{\rm{V}}}_{{\rm{M}}}=-\,{\rm{bk}}/4$$

The period during which 58% of the N, P, and K has accumulated is defined as the N, P, and K (FAP), which begins at *t*_1_ and ends at *t*_2_. During the FAP, Y is linearly correlated with *t*, and the V_T_ is calculated as follows:

4 $${{\rm{V}}}_{{\rm{T}}}={{\rm{Y}}}_{2}-{{\rm{Y}}}_{1}/{t}_{2}-{t}_{1}$$
